# Monthly Summary

**Published:** 1880-07

**Authors:** 


### MONTHLY SUMMARY.

---

*A Nerve Stimulant.*—There are two nerves, says Dr. Brunton, in the *Contemporary Review*, known as the "fifth pair," which are distributed to the skin of the head and to the mucous membrane of the eyes, nose, and mouth. These nerves are closely connected with the heart and vessels, and by stimulating their branches the circulation may be influenced, as in the case of fainting. It is a curious fact that people of all nations are accustomed, when in any difficulty, to stimulate one or another branch of the fifth nerve, and quicken their mental processes. Thus, some persons when puzzled, scratch their heads; others rub their foreheads; and others stroke or rub their beards, thus stimulating the occipital, frontal, or mental branches of these nerves. Many Germans when thinking have a habit of striking their fingers against their noses, and thus stimulating the nasal cutaneous branches, while in other countries some people stimulate the branches distributed to the mucous membrane of the nose by taking snuff.

The late Lord Derby, when translating Homer, was accusetomed to eat brandied cherries. One man will eat figs while composing a leading article; another will suck chocolate cremes; others will smoke cigarettes; and others sip brandy and water. By these means they stimulate the lingual and buccal branches of the fifth nerve, and thus reflexly excite their brains. Alcohol appears to excite the circulation through the brain reflexly from the mouth, and to stimulate the heart reflexly from the stomach, even before it is absorbed into the blood. Shortly after it has been swallowed, however, it is absorbed from the stomach, and passes with the blood to the heart, to the brain and to other parts of the nervous system, upon which it then begins to act directly. Under its influence the heart beats more quickly, the blood circulates more freely and thus the functional powers of the various organs in the body is increased so that the brain may think more rapidly, the muscles act more powerfully, and the stomach digest more easily. But with this exception, the effect of alcohol upon the nervous system may be described as one of progressive paralysis. The higher centres suffer first, and the judgement probably is the first quality to be imparied, and this becomes the more so as the effect of the alcohol progresses, although the other faculties of the mind may remain not only undiminished by the action of the alcohol on the brain, but greatly increased by the general excitement of the circulation. By and by, however, the other parts of the nervous system are successively weakened, the legs fail and the person falls insensible. It is evident, then, that only the first stages of the alcoholic action are at all beneficial, the lattter being as clearly injurious.—*Druggist's Circular.*

---

*Use of Chloroform in Diseases of the Heart.*—On this subject M. Vergely, of Bordeaux, (*La France Medicale,*) remarks that there is a difference of opinion, some asserting that chloroform is very useful, and others that it does harm in affections of the heart. In M. Vergely's memoir, to which M. Dieulafoy has recently drawn the attention of the Societe Medicale des Hospiteaux, three principle points are established.

1. That the existence of heart disease does not contraindicate the use of anæsthetics.

2. That chloroform is a sedative in this class of disease.

3. That it should be used with discretion. In some cases of severe palpitation chloroform may be successfully administered. Also in some cases of dyspnœa and palpitation arising from insufficiency, either alone or conjointly with hypodermic injections of morphia. M. Vergely has also given it without any accident in angina pectoris, and in certain other affections of the heart characterized by dyspnœa and palpitation. From inquiries he has made into the literature of the subject, he concludes that this agent has been employed too timidly and unsystematically. —*Druggists Circular.*

---

*The Setting of Plaster.*—Why does plaster set is a question often asked. This was invested by Dandrin, who examined the chemical and physical changes that occurred during the process of solidification. With the aid of the microscope he observed that burned plaster, in contact with water, assumes a crystalline form; that the water surrounding the crystals takes up in solution considerable sulphate of lime, and that, a portion of this water being evaporated by the heat resulting from the chemical combination, a crystal is formed which determines the crystallization of the whole mass, just as when a crystal of sulphate of soda is dropped into a saturated solution of that salt. It is not, however, until after some time that the mass acquires its maximium hardness, the plaster then containing the required proportion of water, that is, two molecules to one of the sulphate of lime. This amount of water does not lessen by evaporation.

In mixing plaster, only about 12 per cent. of water should be added, as ordinary plaster itself contains about 8 per cent.; but in actual practice the amount used is never less than 33 per cent. This excess is added in order to prevent setting before it can be used. But the effect is injurious, since very porus, slowly drying plasters are produced in this way, which rapidly determine nitrification. To diminish the rapidity of setting is to delay the crystallization. This can be effected by adding gum, gelatine, glycerine, or similar bodies. Inert substances, like sand, for example, on the other hand, simply diminish the solubility of the material without in the least retarding the setting process.

Over-burned plasters may be utilized by mixing them with ordinary plaster, as the crystallization of the latter will extend to the former and occasion the setting of the entire mass. Lime acts favorably on plaster, as it not only increases the rapidity with which it sets, but it gives it additional hardness. Plasters to which 10 per cent. of lime has been added are capable of taking a polish.—*Chemical Gazette*

---

*A Form of "Land Scurvy."*—In the last (February) number of the New Orleans *Medical and Surgical Journal*, a correspondent in Louisiana describes an affection which strongly resembles what is known in Germany as "land scurvy." He states that the part disordered is the gums, which are of a dark red, have a tendency to bleed easily, cleave from the teeth, making them look unnaturally long; the breath is disagreeable and the patient does not seem to suffer much inconvenience from it, except at times when the disease seems to be particularly increased. The patients drink, smoke, chew tobacco, etc. and eat as others do. There is no unusual salivation, but when they bleed it seems to be from a surface, not from any crack or fissure. The parties are not of syphilitic character. It goes locally under the name of "scurvy," and is very difficult to cure. He says he has never cured a case yet, although he has tried acids, natural salts, astringents locally, together with iodide of potash internally.

We have seen such cases occasionally in the Northern States, but have found them to yield readily to chlorate of potash and citric acid, with a varied diet and improved sanitary surroundings.—*Med. and Surg. Reporter.*

---

*A New Narcotic.*—Jamaica dogwood, piscidia erythrina is recommended in the London Pharmaceutical Journal as powerful narcotic, capable of producing sleep and relieving pain in an extraordinary manner. It has been used as an anodyne in toothache, curing the pain when introduced upon a fossil of cotton into the carious tooth. In Brazil it has an established reputarion as nervous sedative. Its actions seem to be over the nerve-centres; it causes sleep without producing the cerebral hyperæmia which succeeds opium and morphia. The sleep is tranquil and refreshing; it soothes bronchial cough and moderates the paroxyism of asthma and nervous coughs. It has been used with ssuccess in chronic hepatitis and obstructions of the liver. —*The Druggist.*

---

*Apparent Death as a Result of Asphyxia.*—Medical journals occasionally inform us of wonderful resuscitations brought about by the persistent use of artificial respiration, but two, lately reported to the Academy of Sciences. Paris, by Dr. Fort, Professor of Anatomy in the Ecole Pratique, are especially noteworthy and teach us to persevere in any efforts we may make to bring signs of life. In the case of a child, three years old, who had already been placed in his shroud, Dr. Fort commenced the use of artificial respiration three and a half hours after apparent death. After four and a half hours steady work, the child was brought back to life. The other case was that of a drowned man, who had been under water twelve minutes before the body was recovered. Artificial respiration was commenced an hour afterwards, and after being kept up for hours, the man was restored to life. The report of the meeting of the Academy does not give the details of the child's case before the appearance of the apparent death, nor what prompted the doctor to commence the artificial respiration.—*L' Union Med.*

